# Systems biology network reveals the correlation between COX-2 expression and Ch 7q copy number alterations in Ch 11q-deleted pediatric neuroblastoma tumors

**DOI:** 10.18632/genesandcancer.225

**Published:** 2022-12-02

**Authors:** Thatyanne Gradowski Farias da Costa do Nascimento, Mateus Eduardo de Oliveira Thomazini, Nilton de França Junior, Lisiane de Castro Poncio, Aline Simoneti Fonseca, Bonald Cavalcante de Figueiredo, Saulo Henrique Weber, Roberto Hirochi Herai, Lucia de Noronha, Luciane R. Cavalli, Bruno César Feltes, Selene Elifio-Esposito

**Affiliations:** ^1^Graduate Program in Health Sciences, School of Medicine and Life Sciences, Pontifícia Universidade Católica do Paraná, Curitiba, Paraná, Brazil; ^2^Biotechnology Undergraduate Program. School of Medicine and Life Sciences, Pontifícia Universidade Católica do Paraná, Curitiba, Paraná, Brazil; ^3^Research Institute Pelé Pequeno Príncipe, Faculdades Pequeno Príncipe, Curitiba, PR, Brazil; ^4^Graduate Program in Animal Science, School of Medicine and Life Sciences, Pontifícia Universidade Católica do Paraná, Curitiba, Paraná, Brazil; ^5^Research Department, Instituto Buko Kaesemodel (IBK), Curitiba, Paraná, Brazil; ^6^Lombardi Comprehensive Cancer Center, Georgetown University, Washington, DC 20007, USA; ^7^Institute of Informatics, Department of Theoretical Informatics, Federal University of Rio Grande do Sul, Porto Alegre, RS, Brazil; ^8^Institute of Biosciences, Department of Biophysics, Federal University of Rio Grande do Sul, Porto Alegre, RS, Brazil

**Keywords:** pediatric cancer, inflammation, chromosomal aberration, interaction network, neuroblastoma

## Abstract

Tumor-associated inflammation and chromosomal aberrations can play crucial roles in cancer development and progression. In neuroblastoma (NB), the enzyme cyclooxygenase-2 (COX-2) is associated with copy number alterations on the long arm of chromosome 11 (Ch 11q), defining an aggressive disease subset. This retrospective study included formalin-fixed paraffin-embedded tumor samples collected from nine patients during diagnosis at the pediatric Pequeno Principe Hospital, Curitiba, PR, Brazil, and post-chemotherapy (CT). COX-2 expression was evaluated using immunohistochemistry and correlated with the genome profile of paired pre- and post-CT samples, determined by array comparative genomic hybridization. A systems biology approach elucidated the *PTGS2* network interaction. The results showed positive correlations between pre-CT Ch 7q gain and COX-2 expression (ρ = 0.825; *p*-value = 0.006) and negative correlations between Ch 7q gain and Ch 11q deletion (ρ = −0.919; *p*-value = 0.0005). Three samples showed Ch 11q deletion and Ch 7q gain. Network analysis identified a direct connection between *CAV-1* (Ch 7q) and COX-2 in NB tumors and highlighted the connection between amplified genes in Ch 7q and deleted ones in 11q. The identification of hub-bottleneck-switch genes provides new biological insights into this connection between NB, tumorigenesis, and inflammation.

## INTRODUCTION

Neuroblastoma (NB) is the most frequent extracranial solid tumor in children, and it accounts for 8–10% of all pediatric cancers [1]. NB tumors originate from neural crest cells, which are primitive progenitors of sympathetic ganglia that can arise anywhere along the sympathetic nervous system [2]. NB presentation differs from other solid tumors because it is highly heterogeneous and ranges from tumors that undergo spontaneous regression to tumors with a highly aggressive profile [1].

*MYCN* oncogene amplification (MNA) is an independent poor prognostic factor significantly associated with unfavorable histological features [3]. Tumor cell ploidy and segmental chromosomal aberrations, which are frequently found in chromosomes 1p, 1q, 3p, 11q, 14q, and 17p, have substantially improved NB risk stratification [3, 4]. Specifically, loss of heterozygosity (LOH) in chromosome 11q (Ch 11q) in nonamplified-*MYCN* (NAMN) was found to be associated with a therapy-resistant metastatic NB subgroup [5] as well as with high activity of the cyclooxygenase (COX)/microsomal prostaglandin E synthase (mPGES)-1/PGE2 pathway [6].

Prostaglandins (PGs) are arachidonic acid-derived chemical mediators of inflammatory response [7] produced by sequential actions of COX-1, COX-2, and specific synthases. Aberrant COX-2 expression are often found in tumor cells by [8], cancer-associated fibroblasts and type-2 tumor-associated macrophages [9]. In the tumor microenvironment, COX-2 decreases the apoptosis of tumor cells by upregulating the expression of the anti-apoptotic protein survivin [10], impairing cell adhesion by downregulating E-cadherin [11], and promoting epithelial to mesenchymal transition (EMT) by upregulating miR-526b expression [12]. Other studies have shown that COX-2 expression can be associated with angiogenesis, tumor cell proliferation, and survival and correlates with invasiveness and resistance to chemotherapeutic drugs in many cancer types [13].

Chemotherapy (CT) can induce changes in residual and relapsed tumors [14], either by the survival of minor clones and their expansion during CT or by the development of genetic alterations in tumor cells that promote their survival and resistance to the same treatment. Recently, cytotoxic therapy has been shown to acutely enhance *COX-2* transcription and PGE2 production in cancer cells, which modifies the tumoral inflammatory response and the efficacy of cytotoxic- and immuno-therapies, although such responses are only observed in tumors with prior activation of the pathway and basal levels of COX-2 mRNA [15]. This finding reinforces the idea that the inflammatory profile and COX-2 expression in the tumor environment prior to treatment may influence the potential therapeutic response. In the current study, we analyzed the COX-2 levels in NB tumor samples and correlated this expression with segmental chromosome aberrations. Using a pipeline of computational systems biology tools, we investigated the direct and indirect connections between *PTGS2* and correlated aberrations to search for new insights on inflammation in the pathophysiology of high-risk NB.

## RESULTS

### COX-2 expression was positively correlated with Ch 7q amplification in pre-CT primary tumors

COX-2 seemed to be randomly expressed in the tumor samples. The highest levels were found in the post-CT samples Pat13, Pat20, and Pat81. Inversely, higher expression was found in the pre-CT samples Pat15 and 42, relative to the post-CT counterparts.

**Figure 1:**
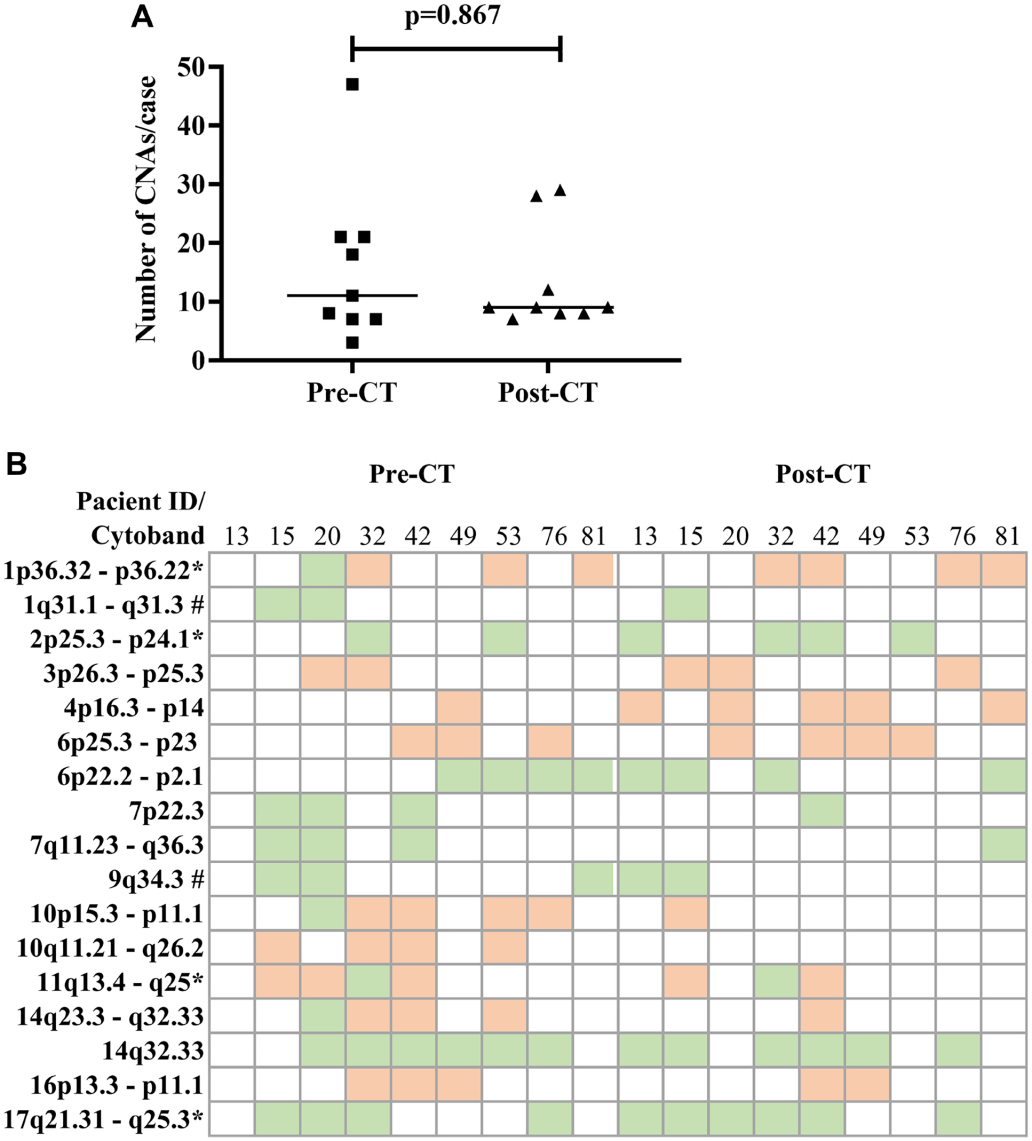
Cytobands with copy number alterations (CNA) in paired NB samples pre-and post-chemotherapy. (**A**) Median of CNAs per case. (**B**) List of CNAs affecting at least three (30%) cases, including those of prognostic importance (^*^) and those involved in the COX-2 pathway or correlated with COX-2 expression (^#^), in pre- and post-treatment samples from each patient. Orange squares indicate losses, and green squares, gains.

An a-CGH analysis of the paired sample sets (*n* = 9) revealed that the median (min-max) of the copy number alterations (CNAs) in each case did not significantly differ between pre-CT [11, 3–47] and post-CT [9, 7–29] (*p*-value = 0.867) (Figure 1A). The most frequent affected cytobands occurring in at least 30% of cases were compared in the paired samples (Figure 1B). The CNA distribution and frequency varied randomly between pre- and post-CT tumor samples. Cytoband 10q11-q26 (loss) was exclusively found in the pre-CT samples. In contrast, the CNA frequency was higher after CT in Ch 2p (44%), 4p (55%), and 17q (66%).

A correlation analysis was performed that included clinical data, copy number alterations, and COX-2 immunoexpression, with correlations at *p* ≤ 0.05 considered significant (Supplementary Table 1). As expected, overall survival (OS) was inversely correlated with age at diagnosis (Spearman ρ = −0.828, *p*-value = 0.042). Positive correlations between COX-2 expression and Ch 7p22.3 gain (ρ = 0.825; *p*-value = 0.006) and Ch 7q11.23-q36.3 gain (ρ = 0.825; *p*-value = 0.006) were found in the pre-CT samples. Also, a strong inverse correlation was observed between Ch 7q11.23-q36.3 gain and Ch 11q13.4-q25 deletion (ρ = −0.919; *p*-value = 0.0005) in pre-CT samples. In the post-CT analysis, a correlation was observed between COX-2 post-CT expression and tumor regression (ρ = 0.760; *p*-value = 0.0028). Therefore, the three patient samples that had the amplification at Ch 7q, the deletion at Ch 11q, and COX-2 expression (Pat15, Pat20, and Pat42) were selected for analysis using systems biology techniques.

### Hub-bottleneck-switches present on Ch 7q and 11q connect these alterations to NB tumors and altered COX-2 expression

**Figure 2:**
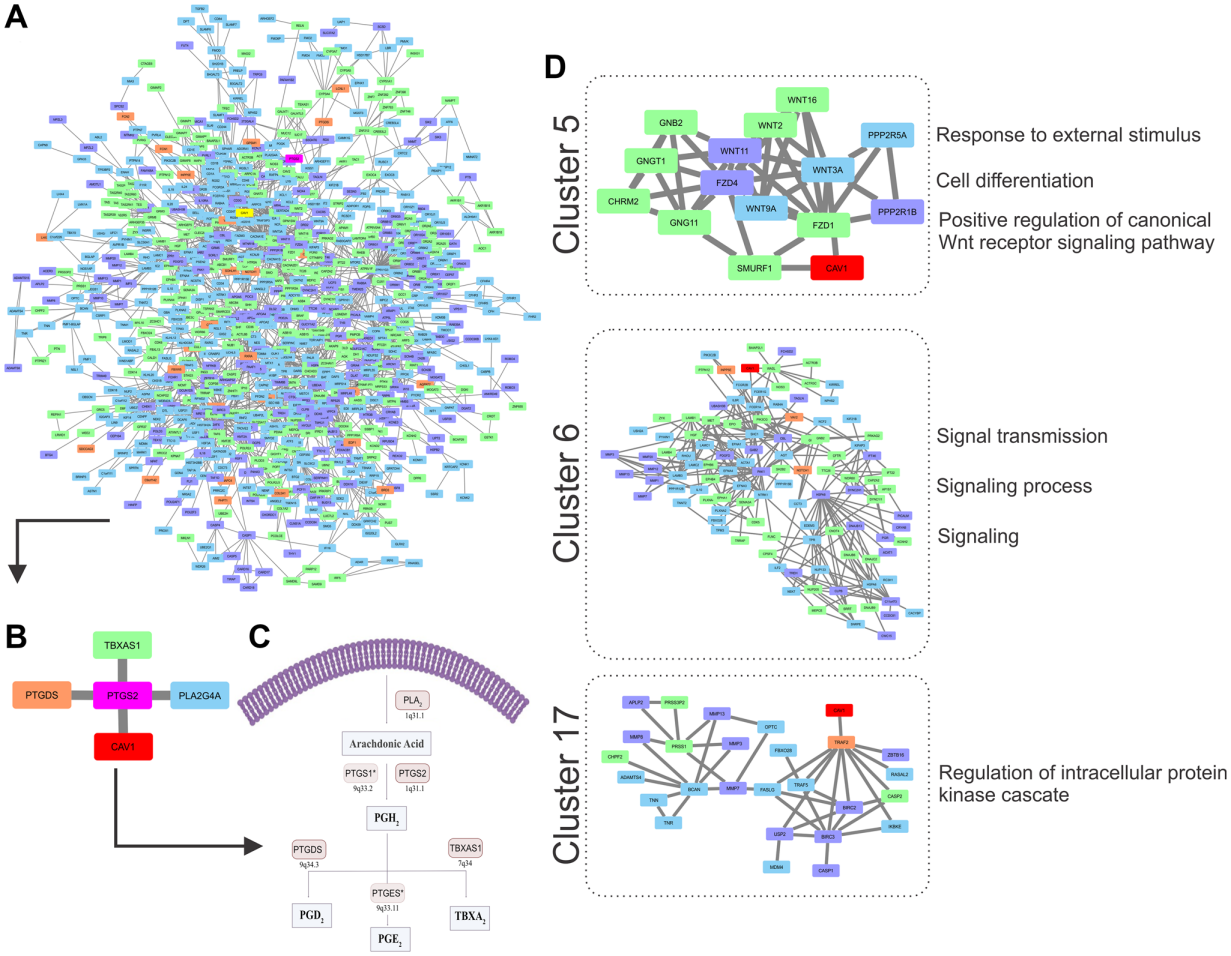
(**A**) PPI network showing the 901 nodes (blue represents Chr1q gain, green Chr 7q gain, orange Chr 9q gain, purple Chr 11q deletion). The *PTGS2* gene is marked in pink and *CAV-1* in yellow. (**B**) *PTGS2* direct connections. (**C**) Prostaglandin 2 (PGE2) pathway showing the main enzymes and the chromosome location of the genes involved (PTGS1* and PTGES* are not included in the PPI network due to their location). (**D**) Three clusters with *CAV-*1 and the gene ontologies (GO) related to *CAV-1* found in which cluster.

Pre-CT samples of Pat15, Pat20, and Pat42 that presented aberrations in Ch 7q11.23-q36.3 and Ch 11q 13.4-q25 (Figure 1) were further investigated using a systems biology approach. Genes located within these cytobands were identified for the construction of a protein-protein interaction (PPI) network. Similarly, protein-coding genes located at Ch 1q21-q44 and Ch 9q34.2-q34.3 were also selected, which resulted in a network with 901 nodes and 2405 edges (Figure 2A). The topological analysis resulted in the identification of 67 hubs, 44 bottlenecks, 71 hub-bottlenecks (HBs), 38 switches, and 107 hub-bottleneck-switches (HBSs) (Supplementary Table 2).

HBS analysis showed that 43% of the 107 nodes belonged to Ch 1q, 34% to Ch 7q, 4% to Ch 9q, and 20% to Ch 11q. These nodes can be considered as having higher topological influence, which can be due to their multiple connections and their control of the network information flow. Among the genes present in the deleted region of Ch 11q, we found DNA damage response (DDR)–associated genes such as homolog A, double-strand break repair nuclease (*MRE11A*), H2A histone family member X (*H2AFX*), and checkpoint kinase 1 (*CHK1*). A master regulator of DDR and cell cycle checkpoint kinase, ataxia-telangiectasia mutated (*ATM*); protein phosphatase 2 scaffold subunit A beta (*PPP2R1B*), which encodes a subunit of the heterotrimeric protein phosphatase 2 (*PP2A*). Embryonic ectoderm development (*EED*) in Ch 11q is part of the polycomb repressive complex 2 (PRC2) together with enhancer of zeste homolog 2 (*EZH2*), which is also an HBS, but is present in Ch 7q.

**Figure 3:**
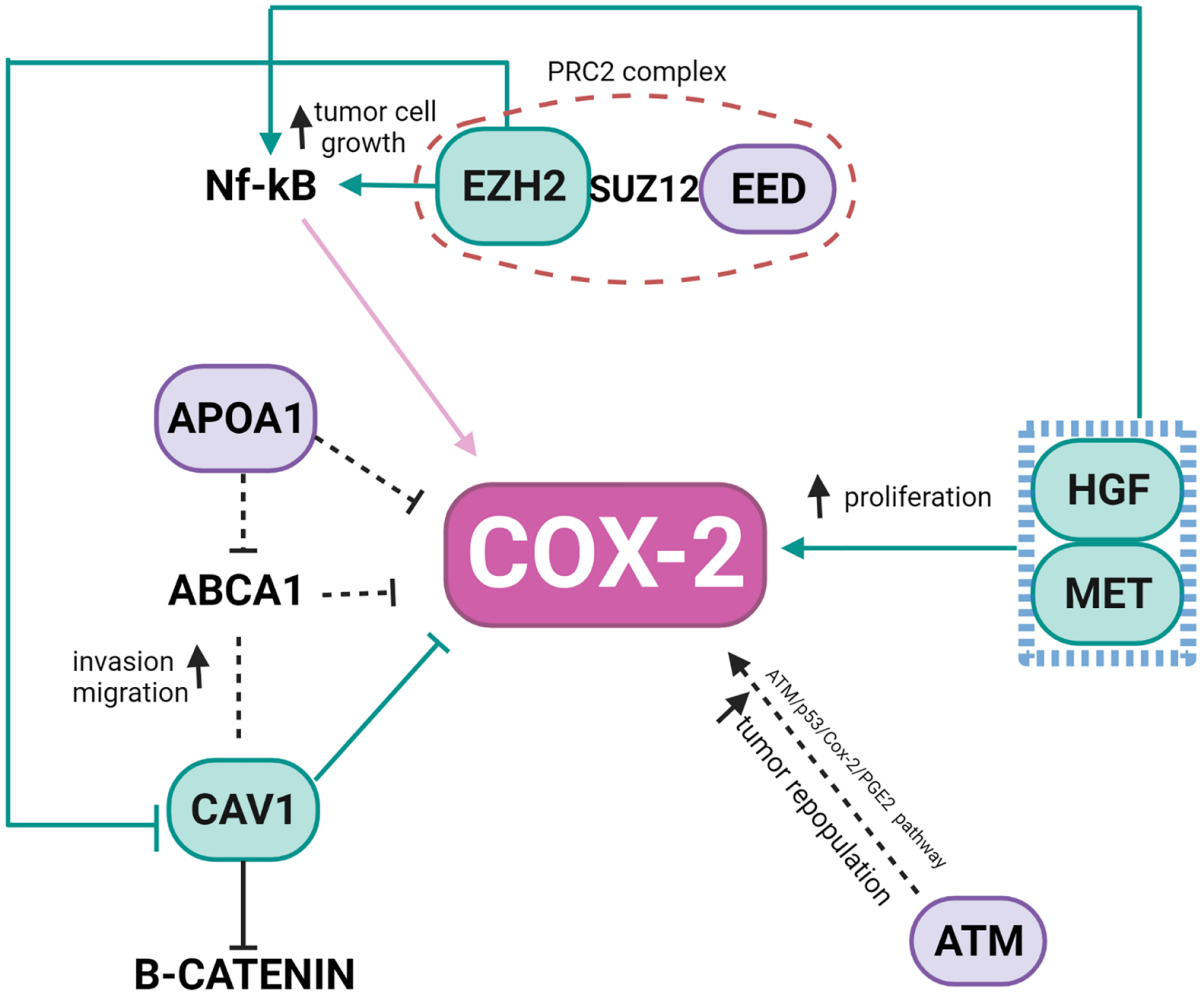
Representation of the interconnection of genes of higher relevance in the network as described previously. It shows the mutual association of Ch11q deleted genes (purple), Ch 7q amplificated genes (green) and COX-2 (pink). Black dotted arrows are representing the gene deleted actions.

In the Ch 7q amplified region, we can highlight the oncogene staphylococcal nuclease and tudor domain containing 1 (*SND1*) as well as components of the SWI/SNF complex such as actin-like 6B (*ACTL6B*), a regulator of chromatin, subfamily D, member 3 (*SMARCD3*) that regulates gene transcription by mobilizing nucleosomes. Also, the proto-oncogene tyrosine–protein kinase Met (*MET*), together with hepatocyte growth factor (*HGF*), plays a role in embryogenesis, EMT, growth, and survival of cancer cells and stimulates metastasis. Gene Ontology (GO) analysis for HBS showed “response to hormone stimulus”, “regulation of phosphorylation”, and “signal transduction” as related processes. Once all the nodes present in Ch 11q are deleted in the patients analyzed, their biological functions are compromised. Hence, we highlight that the interactions in network need to be analyzed considering their absence. Thus, the model shown in Figure 3 was built, showing the direct and indirect interactions found between the highlighted HBS.

### PPI network reveals a direct connection, not yet explored in NB, between COX-2 and CAV-1

*PTGS2* is a bottleneck with direct connections to phospholipase A2 Group IVA (*PLA2G4A*) in Ch 1q, prostaglandin D2 Synthase (*PTGDS*) in Ch 9q, and thromboxane synthase 1 (*TBXAS1*) in Ch 7q, all involved with the COX-2 pathway. Caveolin 1 (*CAV-1*) in Ch 7q was the only direct connection that was not part of the COX-2 pathway (Figure 2A–2C), and it presented topological relevance in the PPI as a HB.

Clustering analysis led to the identification of 17 clusters, and although the *PTGS2* gene was not found in any of them, *CAV-1* was found in clusters 5, 6, and 17 (Figure 2D). After performing a GO analysis, cluster 5 was associated with the terms “response to external stimulus”, “regulation of canonical Wnt receptor signaling pathway”, “cell differentiation”, and “cell communication”. In cluster 6, the GO terms included “signaling processes”, and in cluster 17, the terms included “regulation of intracellular protein kinase cascade” (Figure 2D).

Considering these findings, whether the expression of the proteins COX-2 and CAV-1 would be correlated in the formalin-fixed paraffin-embedded (FFPE) pre- and post-CT paired samples was examined. All samples were positive for CAV-1, but a statistically significant association was not found, possibly due to the small sample size.

Although correlations were found between the clinical and genetic variables, no cytobands with coincidental CNAs were identified among the three patient samples analyzed post-CT; thus, building a network was impossible. All three patients presented normal Ch 7q, and Pt20 did not show Ch 11q deletion post-CT (Figure 1B). Due to the direct connection present in the network, CAV-1 was the only node investigated by immunohistochemistry (IHC).

## DISCUSSION

In the last few decades, the importance of the inflammatory responses in determining disease progression in patients with cancer has become recognized [16, 17] and treatments targeting inflammatory pathways serve as a therapeutic option for NB and other types of cancer. High-risk NB, in particular, the therapy-resistant subset with chromosome 11q-deletion, was suggested to be inflammatory driven and characterized by high activation of the PGE2 pathway with poor treatment response [6]. In the present study, we showed that expression of COX-2, a key enzyme in the pathway, did not significantly differ between pre- and post-CT samples and did not correlate with 11q-deletion. However, a positive correlation was revealed between COX-2 and Ch 7q gain in the pre-CT samples, which in turn inversely correlates with 11q deletions. Our PPI network presented a direct connection of *PTGS2* with *CAV-1* and highlighted topologically relevant nodes in both chromosomes, revealing that the regulation of COX-2 in NB tumors may be based on complex interactions among proteins coded in Ch 7q11.23-q36.3 and Ch 11q13.4-q25 (Figure 3).

CAV-1 is a member of a family of structural proteins, and it regulates inflammatory mediator production [18]. Previous studies have shown COX-2 and CAV-1 colocalization at the plasma membrane of cancer-associated fibroblasts [19], as well as the positive correlation of their expression within plasma membrane caveolae-like structures in lobular breast cancer cells [20]. Functionally, the augmented expression in tumor cells with low basal levels of CAV-1 reduced COX-2 mRNA and protein levels, beta-catenin-Tcf/Lef and COX-2 gene reporter activity, PGE2 production, and cell proliferation [21]. Apolipoprotein A1 (*APOA1*), located in the Ch 11q deleted region, was previously reported as an inhibitor of COX-2 expression in colorectal cancer, which results in the negative regulation of phospholipid-transporting ATPase ABCA1. ABCA1 is significantly overexpressed in patients in advanced stages of colorectal cancer, and its overexpression confers proliferative advantages by regulating CAV-1 stability [22]. *APOA1* is an HBS node in the network, connecting COX-2, CAV-1, and Ch 11q in NB tumors.

The polycomb repressive complex 2 (PRC2) is responsible for an epigenetic role in cancer development, progression, and prognosis. It is formed by the association of *EZH2* (Ch 7q HBS), with two additional proteins, *EED* (Ch 11q HBS) and Suppressor of zeste 12 (*SUZ12*). In our pre-CT data, this complex was not formed once *EED* was deleted. EED is considered a core component that interacts with EZH2 through a WD40 domain, while EZH2 is the catalytic unit of PRC2 [23]. The absence of EED is related to a loss of PRC2 methylation function in embryonic stem cells [24, 25]. In *MYCN*-amplified NB, *EED* knockdown inhibited NB cell proliferation [26], and the amplification of *EZH2* acted to prevent cell differentiation [27]. Usually, high-risk NB patients, after consolidation therapy, undergo myeloablative autologous hematopoietic stem cell transplantation, local radiation, and then immunotherapy with differentiation therapy as the maintenance phase [1]. In breast cancer, EZH2 promotes the expression of nuclear factor-κB (NF-κB) targets and tumor cell growth independent of its histone methyltransferase activity [28]. NF-κB is a transcription factor responsible for the induction of pro-survival genes and several chemokines and cytokines; it is also a key regulator in the production of COX-2 [29]. None of our patients presented a deletion in Ch 7q (Figure 1B), suggesting that the EZH2 may be acting without interference. In gall bladder carcinoma (GBC), EZH2 and COX-2 were chosen as biomarkers and considered future targets in GBC therapy [30]. EZH2 also promotes tumor progression in pancreatic [31] and prostate cancers [32]. Moreover, in prostate cancer, it was found that genomic amplification in the region Ch 7q31-36 could result in downregulating *CAV1,* while overexpressing *EZH2*. This interaction was not a direct connection in our network.

The somatic alterations found in Ch 7q and Ch 11q are complex, and more than one gene may likely be involved in tumorigenesis and progression. Besides the HBS connecting Ch 7q and Ch 11q, there are some that may alone influence COX-inflammation-NB triangle. The HGF/MET complex has been reported to signal migration and/or differentiation of neural crest cell-derived structures [33], via overexpression, amplification, aberrant splicing, or mutations, associated with many cancer types [34, 35]. The HGF/MET complex can also influence COX-2 expression in glioma cells where complex signaling promotes PGE2 release, up-regulating COX-2 expression [36]. *ATM*, another gene absent due to Ch 11q deletion, may play a role in NB and inflammation. An ATM/p53/Cox-2/PGE2 pathway described in non-small cell lung cancer (NSCLC) post-radiotherapy demonstrated that the ATM/p53 cascade increased production of COX-2/PGE2 in the presence of activated caspase-3 [37].

Our study had limitations based on the number of samples, which prevented the possibility of an association between COX-2 expression and clinical aspects of the disease. Nonetheless, the systems biology approach provided a direction and indicated that aberrations in Ch 7q may affect the regulation of COX-2 in Ch 11q-deleted NB tumors. This study also highlights the possible relation between the inflammatory process and cancer through DNA damage and epigenetic changes on important candidates, both Ch 7q and Ch 11q, and their interaction with each other. Further studies are needed to understand the exact mechanisms underlying this phenomenon. Validating these findings may confirm the ability of Ch 7q to interfere with COX-2 expression in NB.

## MATERIALS AND METHODS

### Study design and tumor samples

This is a retrospective study conducted using data and tumor samples from pediatric patients diagnosed with neuroblastoma and treated at the Pequeno Príncipe Pediatric Hospital (HPP), Curitiba, PR, Brazil, between 2004 and 2014. A consecutive series of 76 patients were selected for the study. Of these, nine cases presented paired pre- and post-CT FFPE samples with positive COX-2 immunoexpression and good-quality DNA for analysis a-CGH and were included in the study (Supplementary Figure 1). Ethical approval was obtained from the ethics committee of our institute (approval number 33.573.221), and all patients were kept anonymous. The human samples were analyzed following international and national regulations in accordance with the Declaration of Helsinki.

Clinical data were obtained from the Medical Archives and Statistics Service of HPP, and FFPE blocks were obtained from the HPP Biobank. Each tumor specimen was classified according to the following criteria: (i) age at diagnosis (<18 or ≥18 months); (ii) sex; (iii) Shimada classification [38] (favorable or unfavorable histological features); (iv) INSS-based staging (1–4 or 4S); (v) Children’s oncology group (COG)-risk classification [39]; and (vi) clinical course of disease (alive without disease, relapsed, or deceased). The clinical characteristics of this set of patients along with the percentage of COX-2 in each sample are presented in Table 1. The average age at diagnosis was 26 months, with the ages ranging from 0 to 67 months. All cases had unfavorable Shimada classifications [38] and two cases (22%) presented MNA with >4 *MYCN* copies. In five cases, the adrenal gland was listed as the primary tumor location, while bone marrow infiltration was listed in three. Classification according to the International NB staging system (INSS) [40] showed stage 4 as the most frequent (67%), while according to the COG-risk classification, five cases (56%) were included as high-risk NB [39]. Seven patients (78%) died of the disease.

**Table 1:** NB clinical data of paired samples with COX-2 expression pre- and post-CT

Case ID Clinical parameter	13	15	20	32	42	49	53	76	81
Age at diagnosis (mo)	51	67	56	32	6	1	8	0	16
Shimada histology	unfav	unfav	unfav	unfav	unfav	unfav	unfav	unfav	unfav
*MYCN* amplification	No	No	No	Yes	No	No	Yes	No	No
Primary tumor	Ad	Ad	Ab	Ab	Ad	Ab	Ad	Ad	Rt
BM infiltration	Yes	No	Yes	No	No	No	No	Yes	No
Stage (INSS)	4	4	4	4	4	4	3	4S	2
Risk group	HR	HR	HR	HR	IR	IR	HR	IR	LR
Outcome	DOD	DOD	DOD	DOD	DOD	NED	DOD	DOD	NED
COX-2 (%) pre-CT	1.01	6.02	1.98	1.26	2.79	0.51	0.43	1.03	1.26
COX-2 (%) post-CT	3.52	1.96	6.91	0.53	0.32	0.54	1.96	1.13	8.86

Abbreviations: mo: months; unfav: unfavorable; Ab: abdominal; Ad: adrenal; Rt: retroperitoneal; BM: bone marrow; INSS: International neuroblastoma staging system; HR: high risk; IR: intermediate risk; LR: low risk; DOD: dead of disease; NED: no evidence of disease.

### Tissue microarray construction and immuno-histochemical analysis

The immunohistochemistry assay was preceded by the preparation of multisample paraffin tissue blocks (tissue microarray, TMA). The representative areas of tumors were previously identified and demarcated. Two 4 μm thick cylindrical fragments 0.3 cm in diameter were extracted from the original (donor) blocks and were compiled into new TMA blocks. Sections were analyzed by IHC as previously described [41], with modifications. Antigen retrieval was performed using the BioSB®™ immunoretriever (Santa Bárbara, USA). The TMAs were incubated overnight with a primary rabbit polyclonal anti-COX-2 (1:200; Spring Bioscience, USA), or primary rabbit monoclonal anti-Caveolin-1 (1:200; BioSB, USA). Secondary–horseradish peroxidase (HRP)–conjugated antibody (Reveal Polyvalent HRP-DAB Detection System, Spring Bioscience, USA) was incubated for 30 min at room temperature. Positive (colon cancer specimens) and negative (omitting primary antibodies) controls were run in parallel in each of the reactions. The images were obtained using a Zeiss Axioscan Slide Scanner (Jena, Germany) in high power fields (20× magnification), with a total area of 90,472.78 μm^2^. IHC expression was evaluated through quantitative analyses of cytoplasmic staining images using Image-Pro Plus® software (Rockville, MD, USA) and calculated as a percentage of the ratio of positive staining area per the total area [42].

### MYCN amplification status

The *MYCN* oncogene amplification status was assayed by fluorescence *in situ* hybridization on the TMA slides using a direct commercial probe (Surefish 2p24 *MYCN* 277kb p5; Agilent Technologies Inc., Santa Clara, CA, USA). Briefly, the TMA sections were deparaffinized and treated with HCl (0.2N), followed by proteolytic digestion with pepsin (750 U/ml). Hybridization was performed overnight at 37°C in a humidified chamber. The slides were counterstained with 0.2 μmol DAPI in an antifade solution. Samples were analyzed in a blinded manner by manual counting by two independent investigators (S.E.E. and L.R.C.). Digital images were obtained using a confocal microscope (NIKON Instruments Inc., Tokyo, Japan). *MYCN* was considered amplified in samples with ≥4 positive signals.

### Array–comparative genomic hybridization analysis

DNA copy number analysis was performed using an oligonucleotide a-CGH platform (SurePrint G3 Human CGH Microarray 8x60K; Agilent Technologies Inc., Santa Clara, CA, USA), using a previously established protocol for FFPE samples [43, 44]. DNA was isolated using the standard phenol-chloroform method. Reference DNA was prepared from the peripheral blood of a pool of ten healthy donors [45]. Equal amounts of tumor and reference genomic DNA (1–2 μg) were digested, enzymatically labeled using the SureTag Complete DNA Labeling Kit (Agilent Technologies, Inc., Santa Clara, CA, USA), and hybridized to the arrays. The array data were analyzed with the Feature Extraction v.10.10 software and Agilent CytoGenomics v.3.0 software (Agilent Technologies Inc., Santa Clara, CA, USA) using the ADM-2 algorithm, threshold 6.0, and an aberration filter with a minimum of >3 probes [45]. Copy number gains and losses were defined as previously described [44].

### Systems biology analysis

The protein-coding genes from Ch 1q21.3-q42.2, 7q11.23-q36.3, 9q34.2-34.3, and 11q13.4-q25 were used as inputs to generate a *Homo sapiens* PPI network using the STRING database v.11.0 [46]. All active interaction sources included experiments, databases, co-expression, neighborhood, gene fusion, and co-occurrence, but not text mining. The minimum required interaction score was set at medium confidence (0.400). The PPI data were transferred to the Cytoscape v.3.9 software [47], and the CentiScape 2.2 plug-in [48] was used to select the centralities in the whole network. The degree, betweenness, and eigenvector centralities were calculated for the topological analyses. Degree measures how many direct neighbors are connected to a given node, and nodes with above-average degree values are hubs. A bottleneck is a node with above average betweenness, which significantly influences the network structure. HBs represent a node with above-average degree and betweenness. Finally, the eigenvector centrality assigns a relative score to all the nodes in the network based on the concept that connections to high-scoring nodes highly contribute to the network. High eigenvector denotes switches in the network. Nodes with above-average scores in all three centralities are classified as HBSs and have a key influence in regulating molecular networks [49]. Cluster formation was investigated with the Molecular Complex Detection (MCODE) app [50]. Loops, Haircut, and Fluff were chosen in the network using the advanced options. The cut-off point was delimited as nodes >10 and number of connections >3. The Biological Networks Gene Ontology (BiNGO) plug-in was used to investigate Gene Ontologies 34, with hypergeometric testing and the Bonferroni family-wise correction with a significance level of *p* ≥ 0.05. All non-specific bioprocesses, such as regulation of the biological process and regulation of the metabolic process, were excluded for further analysis considering their lack of biological meaning.

### Statistical analysis

Patient sex, age-related risk, tumor stage, Shimada status, recurrence, death, and clinical follow-up data were distributed in relative frequencies. A correlation analysis of COX-2 expression pre-and post-CT was performed by Student’s *t*-test (paired and unpaired), and CNAs pre- and post-CT were compared by the Wilcoxon signed-rank test. The correlation analysis among clinical data, COX-2 and CAV-1 immunoexpression, and CNAs was performed with a parametric correlation Pearson’s test and nonparametric correlation Spearman’s and Kendall’s Tau-b tests, with a two-tailed analysis. For these analyses, CNAs were categorized as unaltered (0), loss or deletion (−1), and gain or amplification (+1). All statistical analyses were performed using IBM SPSS Statistics (IBM Corp., Version 23.0, Armonk, NY) and GraphPad Prism (version 9.0.0, GraphPad Software, San Diego, CA, USA) software, with *p* < 0.05 considered significant.

## SUPPLEMENTARY MATERIALS





## Abbreviations

a-CGH: Array–comparative genomic hybridization
APOA1: Apolipoprotein A1
ABCA1: Phospholipid-transporting ATPase ABCA1
ACTL6B: Actin Like 6B
ATM: Ataxia Telangiectasia Mutated
BiNGO: Biological networks gene ontology
CAV: Caveolin
CHK1: Checkpoint Kinase 1
CNAs: Copy number alterations
COG: Children’s oncology group
COX: Cyclooxygenases
CT: Chemotherapy
DDR: DNA damage response
EED: Embryonic Ectoderm Development
EMT: Epithelial to mesenchymal transition
EZH2: Enhancer of zeste Homolog 2
FFPE: Formalin-fixed paraffin-embedded
FISH: Fluorescence *in situ* hybridization
GBC: Gall bladder carcinoma
GN: Ganglioneuroma
H2AFX: H2A Histone Family Member X
HB: Hub-bottlenecks
HBS: Hub-bottleneck-switches
HGF: Hepatocyte Growth Factor
HPP: Hospital Pequeno Príncipe
IHC: Immunohistochemistry
INSS: International NB staging system
LOH: Loss of heterozygosity
MCODE: Molecular complex detection
MET: Tyrosine-Protein Kinase Met
MNA: *MYCN* oncogene amplification
mPGES: Microsomal prostaglandin E synthase
MRE11A: Double-Strand Break Repair Nuclease
NAMN: Nonamplified-*MYCN*
NB: Neuroblastoma
NF-κB: Nuclear factor-κB
NSCLC: Non-small cell lung cancer
OS: Overall survival
PG: Prostaglandins
PLA2G4A: Phospholipase A2 Group IVA
PP2A: Heterotrimeric protein phosphatase 2
PPI: Protein-protein interaction
PPP2R1B: Protein Phosphatase 2 Scaffold Subunit A beta
PRC2: Polycomb repressive complex 2
PTGDS: Prostaglandin D2 Synthase
PTGS2: Prostaglandin-endoperoxide synthase 2
SMARCD3: Regulator of Chromatin, Subfamily D, Member 3
SND1: Staphylococcal Nuclease and Tudor Domain Containing 1
SUZ12: Suppressor of zeste 12
TBXAS1: Thromboxane synthase 1
TMA: Tissue microarray
