# Supplementary material for: Systems biology network reveals the correlation between COX-2 expression and Ch 7q copy number alterations in Ch 11q-deleted pediatric neuroblastoma tumors
Source: Genes Cancer. 2022 Dec 2;13:60–71. doi: 10.18632/genesandcancer.225 (PMC9718587; doi:10.18632/genesandcancer.225)
Supplement: Supplementary file 1 [file ganc-13-225-s001.pdf]

Systems biology network reveals the correlation between COX-2 expression and Ch 7q copy number alterations in Ch 11q-deleted pediatric neuroblastoma tumors

SUPPLEMENTARY MATERIALS

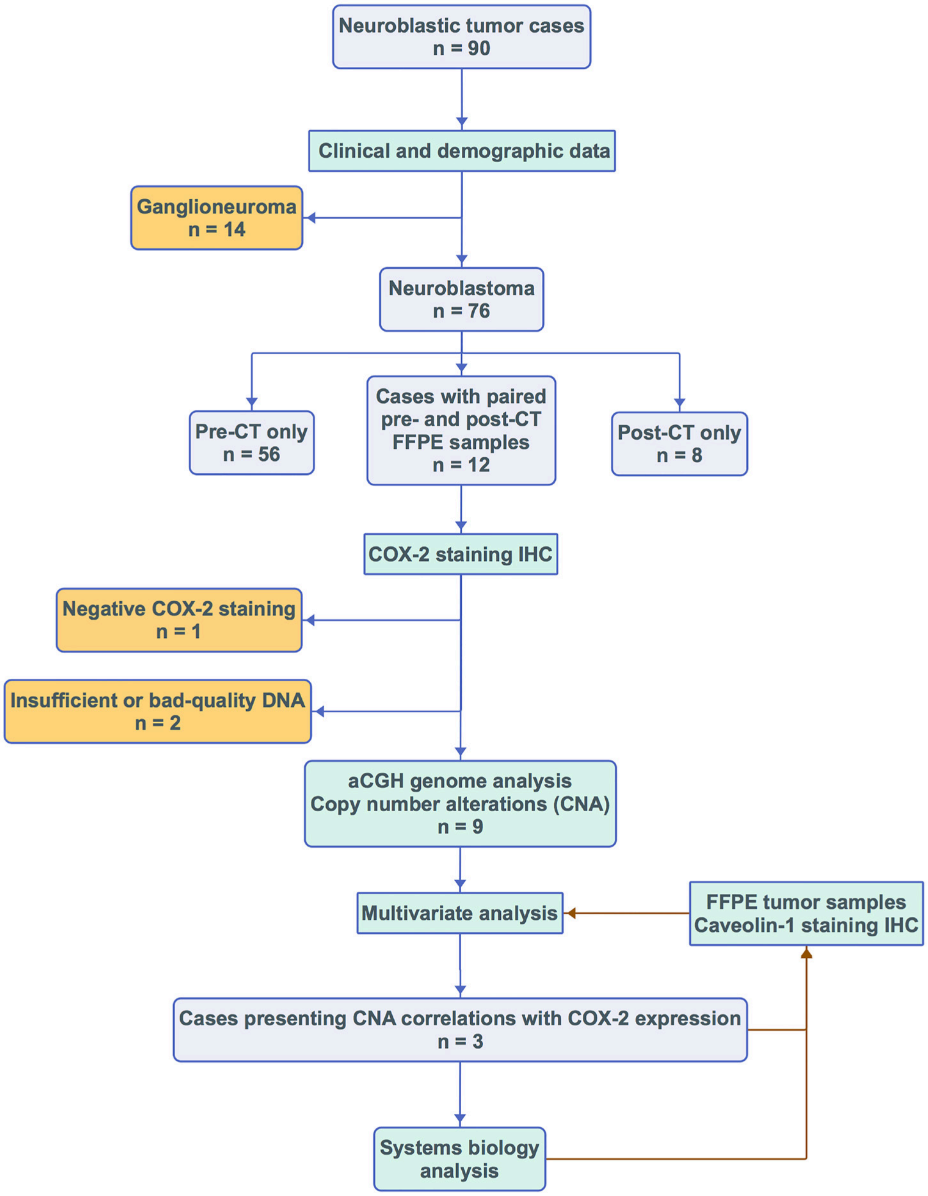

Supplementary Figure 1: Experimental design. Ninety patients treated for neuroblastic tumors in the Pequeno Principe Pediatric Hospital were included in the study. Clinical and demographic data were obtained from medical records, uncovering 76 cases of neuroblastoma. FFPE tumor samples collected in diagnosis and post-chemotherapy were stained for COX-2 by IHC. The genome profile of cases with paired pre- and post-CT COX-2-positive samples were determined by a-CGH and analyzed by statistics multivariate tests. Three cases presented chromosomal copy number alterations correlated with COX-2 expression and segmental chromosomal alterations were deeply analyzed by systems biology approaches. Notes: FFPE, formalin-fixed paraffin-embedded; IHC, immunohistochemistry; CNA, copy number alternations.

Supplementary Table 1: Correlation analysis between clinical data, chromosomal alterations, and immunoexpression of COX-2 and CAV-1. See Supplementary Table 1

Supplementary Table 2: Network topological analysis

HBS	HB	HUB	Bottleneck	Switch
ACTA1	APOA4	ATP5L	ACAT1	ARHGEF12
ACTL6B	ARCN1	ATP6V0E2	ADAMTS4	ATXN7L1
ADCY10	ATP5J2	ATP6V1G3	ADAR	BCL9L
AGK	BCAN	C7orf60	AKR1B1	CD36
AGT	BET1	CHCHD3	ALDH9A1	CDC73
AKAP9	BIRC3	COPG2	ANKRD49	CENPL
APOA1	C11orf73	DOCK4	AVPR1B	CXCR5
APOA2	CAPZA2	DYNC2H1	BRINP2	EIF2D
ARF1	CASP1	EPHB6	C4BPA	FCGR3B
ARPC1B	CAV1	GCC1	CADM3	FLI1
ARPC5	CLPB	GIMAP4	CASP2	GON4L
ARRB1	CNIH3	GIMAP6	CD244	GPR161
ASPM	CNOT4	GRIK4	CUX1	GPSM1
ATM	CNTN2	HYOU1	DARS2	H3F3A
BRAF	COG2	LAMTOR4	ENAH	HIST3H2BB
CACNA1S	COG5	MMP10	F11R	KCNJ5
CBL	COPA	MRPL48	FDPS	MED17
CCT3	CPSF4	MUC12	FMOD	MEF2D
CD247	CRP	NDUFB2	GBA	MTNR1B
CD3D	CRTC2	NDUFC2	GLUL	NFRKB
CD3E	CTSC	NDUFC2-KCTD14	GNPAT	NR5A2
CD3G	CWC15	NUP133	GPC2	OPN1SW
CD48	CYP3A4	PRSS1	HSD11B1	P2RY2
CDK5	CYP51A1	RHEB	HSD17B7	PARP1
CFTR	DAP3	SCN3B	IL18	PDC
CHEK1	DDX6	SDHC	KCNH2	PICALM
CKS1B	DIEXF	STX6	LHX3	PYGO2
COPS6	DLAT	XCL1	MTPN	RGSL1
CUL1	DLD		NNMT	RSF1
DCUN1D5	DYNC1I1	MMP13	ORC5	SNAPC4
DDX10	EDEM3	DNAJC2	PAFAH1B2	TADA1
DHX9	GALNT2	ATP6V1F	PGR	TAF1A
DTL	GRM8	B3GNT6	PTGS2	TAF1D
EED	IARS2	SRP9	PVRIG	TAF5L
EPHA1	IFI16	NRCAM	RAB13	UBAP2L
EPRS	IL10	TMEM126A	SAMD9L	USF1
EZH2	KIFAP3	WNT2	SC5D	VIPR2
FCER1G	LAMTOR2	SDE2	SH2D1B	WDR42A
FCGR2A	MMP1	GIMAP7	SPCS2	
FCGR3A	MMP3	CD55	STT3A	
FZD4	MMP7	F5	TAGLN	

GAB2
GNAI1
GNB2
GNG11
GNGT1
GRM3
H2AFX
HGF
HIST3H3
HSPA8
HTR5A
IL10RA
IL6R
ILF2
KCNJ9
KDM7A
LAMB1
LAMB3
LAMC2
LEP
LMNA
MCM7
MET
MNDA
MRE11A
MRPL24
MRPS14
MRPS2
NCAPG2
NCF2
NOTCH1
NTRK1
NUF2
PAK1
PIK3CG
PLA2G4A
PLOD3
POLR2J
POU2F1
PPP2R1B
PPP2R5A
PRKAG2
PRKAR2B
PSMC2
PTPRC
RBBP5

MUC1
NDUFA5
NDUFS2
NVL
PDIA4
PKLR
PMPCB
PON1
PPP1R3A
RAB6A
RBM28
SEC16A
SEC16B
SHFM1
SHH
SPTA1
SRPK2
SSBP1
ST3GAL4
TMEM126B
TOMM40L
TPM3
TRAF2
TREH
UBQLN4
VAMP4
YAP1
ZNF212
ZW10
ZYX

XCL2
BUD31
MUC17
MRPS33
COL1A2
ARF5
NOM1
WNT16
GPATCH4
NME7
BIRC2
APOC3

GIMAP5
WDR60

SCN2B
SDHD

MRPL55
NUP205
SCN4B
INTS3
COL5A1
DNAJB13
ASB4
EPHB4
SERPINC1
GIMAP8
WNT9A

TNR
UBE2H
ZPR1

RC3H1
RFWD2
RGS18
RPS25
RPS27
RPS3
RXRA
SHC1
SMO
SMURF1
SND1
SNRPE
TPR
TRAF3IP3
TRIM24
TRRAP
UCHL5
WASL
WDR5
ZC3HAV1

List of HBS, HB, B, HUB, Switches.
